# Effect of combined aspirin and statin therapy on mortality reduction in sepsis-induced myocardial injury 

**DOI:** 10.3389/fphar.2026.1839835

**Published:** 2026-06-09

**Authors:** Yunzhang Zhao, Xuan Zhang, Shuai Mao, Huiyi Liu, Chao Lv, Yanzhu Yao, Yuyan Wang, Yifan Wang, Shengshu Wang, Yong Xu, Tong Yin

**Affiliations:** 1 Institute of Geriatrics, National Clinical Research Center for Geriatric Diseases, Second Medical Center of Chinese PLA General Hospital, Beijing, China; 2 Medical School of Chinese PLA, Chinese PLA General Hospital, Beijing, China; 3 Senior Department of Cardiology, The Sixth Medical Center of PLA General Hospital, Beijing, China; 4 Department of Medical Innovation Research, Chinese PLA General Hospital, Beijing, China

**Keywords:** aspirin, combined therapy, mortality, sepsis-induced myocardial injury, statin

## Abstract

**Introduction:**

Sepsis-induced myocardial injury (SIMI) is a severe complication with high mortality and limited effective treatments. Although combined aspirin and statin therapy is cardioprotective in coronary artery disease, its impact on SIMI outcomes remains unclear.

**Methods:**

Using the MIMIC-IV database, we evaluated the association of combined aspirin and statin therapy with 28-day, 90-day, and 1-year mortality in SIMI patients using multivariable Cox regression and Kaplan-Meier analysis. Robustness was assessed via floating absolute risk analysis. External validation was performed in an independent ICU cohort.

**Results:**

Among 1,884 SIMI patients, 119 received combination therapy. Compared to those without aspirin or statin, combination therapy was associated with significantly lower mortality at 28 days (11.8% vs. 41.9%; HR = 0.22), 90 days (15.1% vs. 44.8%; HR = 0.23), and 1 year (29.4% vs. 50.9%; HR = 0.38) (all *P* < 0.001). The combination consistently showed the greatest risk reduction. Subgroup analysis confirmed consistent benefits. External validation (n = 4,002) confirmed a lower 28-day mortality risk with combination therapy (HR = 0.48; *P* < 0.001).

**Conclusion:**

Combined aspirin and statin therapy is associated with reduced mortality and improved outcomes in SIMI patients.

## Introduction

Sepsis and septic shock constitute predominant causes of global intensive care unit (ICU) mortality, exhibiting substantial morbidity and mortality burdens (Singer et al., 2016). Within this clinical landscape, sepsis-induced myocardial injury (SIMI) represents a common yet severe complication characterized by transient myocardial suppression that profoundly exacerbates patients' prognosis (Kakihana et al., 2016). Severe myocardial dysfunction of SIMI is associated with a mortality rate potentially exceeding 80% in patients with sepsis, particularly in those with septic shock (Artucio et al., 1989). Timely reversal of SIMI through early intervention necessitates prioritized cardiac protection strategies. Current guidelines demonstrate that the management of SIMI remains predominantly rooted in supportive care and systemic approaches, whereas a critical therapeutic gap persists in pharmacotherapies aimed at mitigating myocardial injury (Aissaoui et al., 2025). Therefore, developing novel treatment strategies to improve SIMI outcomes presents an urgent clinical priority.

Emerging data suggest that cardiovascular medications, including statin and aspirin, may confer benefits in sepsis (Chen et al., 2022; Morel et al., 2017; Wang et al., 2023; Sun et al., 2024). Statin therapy has been shown to reduce mortality in sepsis via pleiotropic effects, including anti-inflammatory activity, immunomodulation, and endothelial protection (Oesterle et al., 2017). Aspirin attenuates the development of disseminated intravascular coagulation (DIC) by inhibiting microvascular thrombosis and improving tissue perfusion (Wang et al., 2018). Theoretically, combination therapy with statin and aspirin could synergistically suppress sepsis-related myocardial inflammatory cascades, enhance myocardial microcirculatory perfusion, and mitigate cardiomyocyte damage, which could help provide comprehensive SIMI protection. Given the established cardioprotective effect of co-administration of aspirin and statin in patients with coronary artery disease (CAD) (Golub et al., 2023), we hypothesized that the combination therapy could benefit patients with SIMI.

Therefore, the present study aims to investigate the association between the co-administration of aspirin and statin and the risk of mortality in patients with SIMI by analyzing data from the Medical Information Mart for Intensive Care IV (MIMIC-IV) database.

## Methods

### Source of the data

The current investigation performed a retrospective review using the MIMIC-IV database. MIMIC-IV is a large, publicly available repository of de-identified clinical data from over 50,000 ICU admissions at the Beth Israel Deaconess Medical Center (BIDMC) between 2008 and 2022 (Johnson et al., 2023). Requirement for informed consent was waived due to the retrospective and de-identified nature of the data. One of the investigators completed the Collaborative Institutional Training Initiative (CITI) program for human research ethics (certification ID: 14400803 for author Huiyi Liu) and signed a data use agreement prior to accessing the database.

### Study population

The inclusion criteria for participants are defined as follows: age ≥18 years, a confirmed diagnosis of sepsis (Singer et al., 2016), fulfillment of the SIMI criteria within 24 h of ICU admission, and an ICU stay of more than 24 h but no longer than 100 days. Cardiac troponin T (cTnT) was measured on the day of ICU admission, with the highest value obtained that day used for assessing SIMI. The 99th percentile of upper reference limit value for cTnT is 0.01 ng/mL in this center, and SIMI was defined as the cTnT within 24 h > 0.01 ng/mL in this study (Hollenberg and Singer, 2021; Vallabhajosyula et al., 2017; Maeder et al., 2006). We excluded diseases that directly or indirectly lead to abnormal release of cTnT, including acute coronary syndrome (ACS), cardiomyopathy, myocarditis, valvular heart disease, endocarditis, pericarditis, chronic obstructive pulmonary disease (COPD), chronic heart failure (CHF), cardiac surgery before ICU admission, cardiac arrest, and a history of severe tachycardia (supraventricular tachycardia, ventricular tachycardia, ventricular fibrillation, ventricular flutter). In addition, pregnant women and patients with congenital coagulation disorders such as hemophilia, hereditary fibrinogenopenia, and hereditary coagulation factor deficiency were also excluded.

### Data collection

The study data from the MIMIC-IV database was extracted via Structured Query Language (SQL) in PostgreSQL Administration Tool (pgAdmin) software. We collected a comprehensive set of variables recorded within the first 24 h of each patient’s ICU admission. The analyzed variables encompassed demographic characteristics (age, gender and ethnicity); comorbidities including hypertension, diabetes mellitus, CAD, pneumonia, stroke, and cancer, identified using International Classification of Diseases (ICD) codes; and critical illness severity scores, including the Sequential Organ Failure Assessment (SOFA) score and Simplified Acute Physiology Score II (SAPSII). We also documented key interventions, such as mechanical ventilation and continuous renal replacement therapy (CRRT). Pertinent laboratory parameters were extracted and grouped by physiological system, covering: hematological indices (cardiac troponin T [cTnT], white blood cell count [WBC], red blood cell count [RBC], and platelet count [PLT]); coagulation profiles (prothrombin time [PT], activated partial thromboplastin time [PTT] and international normalized ratio [INR]); metabolic and oxygenation markers (lactate); liver function index (alanine aminotransferase [ALT] and aspartate aminotransferase [AST]); renal function indicators (blood urea nitrogen [BUN] and serum creatinine [SCR]); usage of medication (angiotensin-converting enzyme inhibitors [ACEIs], angiotensin II receptor blockers [ARBs], beta-blockers [β-blockers], and anticoagulant agents). All covariates were extracted from measurements recorded within the first 24 h of ICU admission and prior to the first administration of aspirin or statin during the ICU stay, unless otherwise specified. This includes SOFA score, SAPS II score, lactate level, use of mechanical ventilation, and use of CRRT, all of which reflect baseline status at the time of SIMI diagnosis.

### Exposure and outcomes of this study

Exposure was defined as the use of aspirin and statin at any time during the ICU admission. The primary outcome was defined as 28-day all-cause mortality, and the secondary outcomes defined as 90-day or 1-year all-cause mortality. Survival time for all outcomes was calculated from the index date of the diagnosis of SIMI.

### Data cleaning and handling of missing data

Duplicate ICU records were removed, logical consistency of dates was verified, and biologically implausible values for continuous variables were set to missing. All cleaning procedures were performed using PostgreSQL and R software version 4.2.0.

For all clinical and laboratory variables, patients with a missing value rate exceeding 5% for a given variable were excluded from analyses involving that variable. For variables with a missing rate ≤5%, imputation was performed as follows: categorical variables were imputed using the mode, while continuous variables were imputed using the mean of non-missing values within the same treatment group.

### Propensity score matching (PSM)

To minimize confounding by indication, we performed 1:1 nearest neighbor propensity score matching without replacement using a caliper of 0.2. The propensity score model included baseline covariates that differed significantly between comparison groups (*P* < 0.05) based on two sample t tests for continuous variables and chi square tests for categorical variables. The matching variables were: for combination therapy versus nonusers: age, diabetes mellitus, stroke, coronary artery disease, cancer, liver cirrhosis, SOFA, SAPS II, lactate, and white blood cell count; for aspirin versus nonusers: age, diabetes mellitus, pneumonia, stroke, coronary artery disease, liver cirrhosis, SOFA, SAPS II, mechanical ventilation, continuous renal replacement therapy, lactate, creatinine, and white blood cell count; for statin versus nonusers: age, diabetes mellitus, cancer, liver cirrhosis, SOFA, SAPS II, mechanical ventilation, and lactate; for combination therapy versus aspirin: age, pneumonia, coronary artery disease, and cancer; and for combination therapy versus statin: coronary artery disease alone. Covariate balance after propensity score matching was assessed using standardized mean differences (SMD). Consistent with common practice in observational studies, SMD <0.2 was considered acceptable balance, while SMD between 0.1 and 0.2 indicated mild residual imbalance.

### Statistical analysis

Patients were divided into four groups according to aspirin and statin use in the ICU: non-users, aspirin monotherapy, statin monotherapy, and combined therapy. For continuous variables with normal distributions, data are presented as mean ± standard deviation (SD). Non-normally distributed continuous variables are expressed as median (interquartile range). Categorical variables were compared using Pearson’s chi-square test, while continuous variables were analyzed with independent samples t-tests or Wilcoxon rank-sum tests as appropriate.

Covariate selection was primarily guided by the DAG (Supplementary Figure S1) and clinical knowledge. Univariable analyses (*P* < 0.05) were used only as a supplementary check for variables not captured by the DAG. The final multivariable Cox models were adjusted for the following baseline confounders identified from the DAG: age, gender, BMI, hypertension, diabetes mellitus, pneumonia, stroke, coronary artery disease, cancer, mechanical ventilation, CRRT, SOFA score, SAPS II score, and lactate level. We assessed multicollinearity among the covariates included in the multivariable Cox regression model by calculating variance inflation factors. A variance inflation factor exceeding five was considered to indicate problematic collinearity. Hazard ratios (HR) with corresponding 95% confidence intervals (CI) are reported for each variable. The proportional hazards (PH) assumption for the Cox models was tested using Schoenfeld residuals. Variables with a global test *P* < 0.05 were considered to violate the PH assumption. To evaluate sensitivity to unmeasured confounding, we calculated E-values for all primary and secondary comparisons.

To account for differences arising from imbalanced sample sizes between groups, floating absolute risk (FAR) analysis was employed to estimate the HR and 95% CI for each comparison (Lin et al., 2024). Kaplan-Meier analysis was used to calculate the 28-day, 90-day, and 1-year survival probabilities for patients. To address confounding by indication, we performed inverse probability of treatment weighting (IPTW) using multinomial logistic regression. The propensity score model included 14 baseline covariates: age, gender, diabetes mellitus, pneumonia, stroke, coronary artery disease, cancer, SOFA score, SAPS II score, ventilation, CRRT, lactate level, SCR and WBC. Missing data for BMI (40.7%) and lactate (17.0%) were handled using multiple imputation with 10 imputed datasets. The average treatment effect (ATE) was estimated. Weighted Cox proportional hazards models were used to estimate HR with 95% CI for 28-day, 90-day, and 1-year mortality. Balance was assessed using SMD, with SMD <0.1 indicating adequate balance. IPTW-weighted Kaplan-Meier curves with weighted log-rank tests were generated to visualize survival differences across treatment groups. Subgroup analyses stratified by clinical variables were performed using multivariable Cox regression models to investigate the effect of combined aspirin and statin therapy across different subgroups of patients. The P value for the interaction was calculated by including a multiplicative interaction term in the Cox model and assessed using the Wald test.

### External validation

We performed an external validation using data from the Chinese PLA General Hospital, comprising ICU patients with SIMI from January 2020 to October 2025. The external validation was approved by the ethics committee. A multivariate Cox regression model was employed to examine the relationship between aspirin and statin combination therapy and the 28-day mortality.

## Results

### Baseline characteristics of patients with SIMI

**FIGURE 1:**
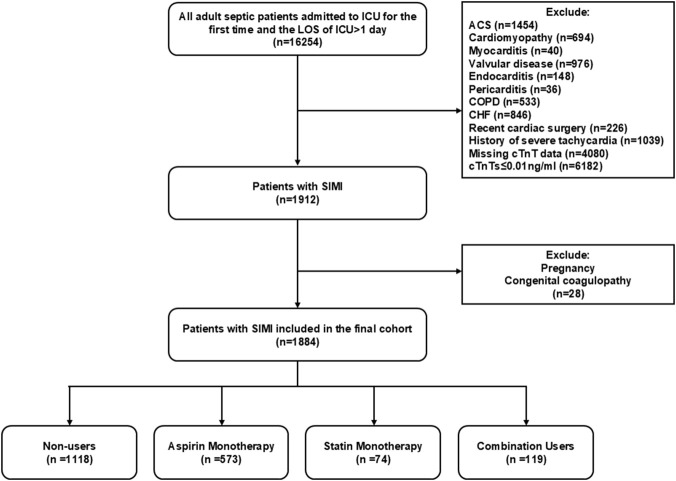
Flowchart of patients’ recruitment. ICU: Intensive Care Unit; LOS: Length of Stay; SIMI: Sepsis-Induced Myocardial Injury; cTnT: Cardiac Troponin T; ACS: Acute Coronary Syndrome; COPD: Chronic Obstructive Pulmonary Disease; CHF: Chronic Heart Failure.

Of the 1,884 patients with SIMI included in the final analysis, 1,118 (59.34%) were non-users, 573 (30.41%) received aspirin monotherapy, 74 (3.93%) received statin monotherapy, and 119 (6.32%) received combined therapy (Figure 1). The baseline characteristics of the study population are summarized in Table 1.

**TABLE 1:** Baseline characteristics of SIMI (n = 1,884).

Characteristics	Non-users (n = 1,118)	Aspirin monotherapy (n = 573)	Statin monotherapy (n = 74)	Combined therapy (n = 119)	P-value
Age, mean (SD)	65 (17.56)	70.38 (14.82)	73.31 (10.25)	74.61 (11.00)	<0.001
Gender (%)	​	​	​	​	0.803
Female	485 (43.38%)	255 (44.50%)	31 (41.89%)	55 (46.22%)	​
Male	633 (56.62%)	318 (55.50%)	43 (58.11%)	64 (53.78%)	​
Ethnicity (%)	​	​	​	​	0.068
White	639 (57.16%)	339 (59.16%)	49 (66.22%)	77 (64.71%)	​
Black	109 (9.75%)	64 (11.17%)	6 (8.11%)	13 (10.92%)	​
Other	370 (33.09%)	170 (29.67%)	19 (25.68%)	29 (24.67%)	​
Vital signs on admission					
HR (bpm)	96.24 (21.91)	89.31 (20.26)	91.47 (21.59)	83.72 (20.93)	<0.001
SBP (mmHg)	130.82 (36.82)	124.40 (27.39)	116.50 (25.66)	125.36 (29.19)	<0.001
DBP (mmHg)	68.25 (20.09)	69.16 (21.53)	66.51 (17.60)	64.46 (17.92)	0.093
Body temperature (◦C)	36.42 (3.93)	36.53 (3.56)	36.13 (6.40)	36.72 (0.87)	0.535
RR (bpm)	21.03 (7.35)	20.13 (5.89)	20.62 (5.91)	18.56 (5.81)	<0.001
SpO2 (%)	96.46 (5.37)	96.86 (4.18)	97.50 (2.94)	97.30 (3.50)	0.009
Comorbidity (%)					
Hypertension	641 (57.33%)	404 (70.51%)	50 (67.57%)	84 (70.59%)	<0.001
DM	304 (27.19%)	211 (36.82%)	30 (40.54%)	50 (42.02%)	<0.001
CAD	130 (11.63%)	204 (35.60%)	14 (18.92%)	62 (52.10%)	<0.001
Pneumonia	476 (42.58%)	280 (48.87%)	27 (36.49%)	44 (36.97%)	0.002
Stroke	90 (8.05%)	73 (12.74%)	11 (14.86%)	16 (13.45%)	0.003
Cancer	160 (14.31%)	46 (8.03%)	4 (5.41%)	10 (8.40%)	<0.001
Laboratory examination					
WBC count (10^9^/L)	14.43 (9.80)	13.54 (7.18)	14.12 (8.28)	12.24 (6.25)	0.003
RBC count (10^12^/L)	3.52 (0.75)	3.63 (0.71)	3.49 (0.63)	3.54 (0.67)	0.067
PLT (10^9^/L)	191.02 (108.45)	208.00 (104.37)	226.97 (119.40)	200.47 (115.27)	<0.001
PT (s)	17.83 (10.15)	15.62 (6.66)	16.01 (4.82)	14.90 (4.65)	<0.001
PTT (s)	40.64 (20.81)	40.69 (22.14)	34.20 (16.18)	37.45 (18.03)	0.022
INR	1.64 (0.98)	1.44 (0.81)	1.47 (0.51)	1.36 (0.48)	<0.001
CRP (mg/L)	75.25 (34.70-145.23)	80.50 (27.20-159.60)	49.20 (35.42-86.28)	74.65 (52.25-107.95)	<0.001
ALT (U/L)	47.50 (22.00-153.00)	31.00 (18.00-85.33)	24.00 (15.00-37.00)	33.00 (19.00-65.67)	<0.001
AST (U/L)	80.25 (37.00-254.50)	52.00 (28.00-149.62)	31.00 (22.00-85.00)	43.00 (29.00-110.62)	<0.001
BUN (mg/dL)	26.75 (18.00-45.35)	27.00 (17.00-44.00)	27.84 (17.69-40.75)	26.00 (17.50-42.20)	0.788
SCR (mg/dL)	1.40 (0.94-2.40)	1.30 (0.87-2.10)	1.35 (0.91-1.98)	1.25 (0.88-1.86)	0.132
Lac (mmol/L)	2.30 (1.50-3.83)	1.85 (1.30-2.86)	1.80 (1.31-2.85)	1.80 (1.30-2.66)	<0.001
Critical assessment on admission					
SOFA score	8.00 (5.00-11.00)	6.00 (4.00-9.00)	6.00 (4.00-8.00)	6.00 (3.00-8.50)	<0.001
SAPS II score	45.00 (35.00-56.00)	41.00 (34.00-52.00)	41.50 (37.25-49.00)	42.00 (36.00-51.00)	<0.001
Treatment (%)					
Ventilation	756 (67.62%)	345 (60.21%)	42 (56.76%)	76 (63.87%)	0.008
CRRT	143 (12.79%)	46 (8.03%)	4 (5.41%)	10 (8.40%)	0.003
Medication, n (%)					
ACEI	60 (5.37%)	83 (14.49%)	11 (14.86%)	21 (17.65%)	<0.001
ARB	30 (2.68%)	29 (5.06%)	5 (6.76%)	7 (5.88%)	0.001
Beta-blockers	482 (43.11%)	393 (68.59%)	41 (55.41%)	85 (71.43%)	<0.001
Clopidogrel	3 (0.25%)	10 (1.75%)	0 (0.00%)	1 (0.84%)	0.003
Low molecular weight heparin	57 (4.67%)	31 (5.41%)	5 (6.76%)	8 (6.72%)	0.409
Oral anticoagulant	47 (4.20%)	36 (6.28%)	7 (9.46%)	10 (8.40%)	0.010

HR, heart rate; SBP, systolic blood pressure; DBP, diastolic blood pressure; RR, respiratory rate; SpO_2_, oxygen saturation; DM, diabetes mellitus; CAD, coronary artery disease; WBC, white blood cell count; RBC, red blood cell count; PLT, platelet count; PT, prothrombin time; PTT, partial thromboplastin time; INR, international normalized ratio; CRP, C-Reactive Protein; ALT, alanine aminotransferase; AST, aspartate aminotransferase; BUN, blood urea nitrogen; SCR, serum creatinine; Lac, Lactate; SOFA, sequential organ failure assessment; SAPS II, Simplified Acute Physiology Score II; CRRT, continuous renal replacement therapy; ACEI, Angiotensin-Converting Enzyme Inhibitor; ARB, Angiotensin II, receptor blocker.

Significant differences across the four groups were observed for multiple variables (*P* < 0.05). These included age; vital signs at admission (heart rate, systolic blood pressure, respiratory rate, SpO_2_); comorbidities (hypertension, diabetes, CAD, pneumonia, stroke, cancer); laboratory parameters (WBC, PLT, PT, PTT, INR, CRP, ALT, AST, lactate); critical illness severity scores (SOFA, SAPS II); and the use of vasopressors, CRRT, ACEIs, ARBs, beta-blockers, clopidogrel, and oral anticoagulants.

After PSM, we successfully matched 119 combination users to 119 non-users, 511 aspirin monotherapy to 511 non-users, 74 statin monotherapy to 74 non-users, 117 combination users to 117 aspirin monotherapy, and 71 combination users to 71 statin monotherapy. Balance was achieved for all covariates in each matched cohort, as evidenced by non-significant differences between groups (all *P* > 0.05; Supplementary Tables S4–S8).

### Association between co-administration of aspirin and statin and survival outcomes

**FIGURE 2:**
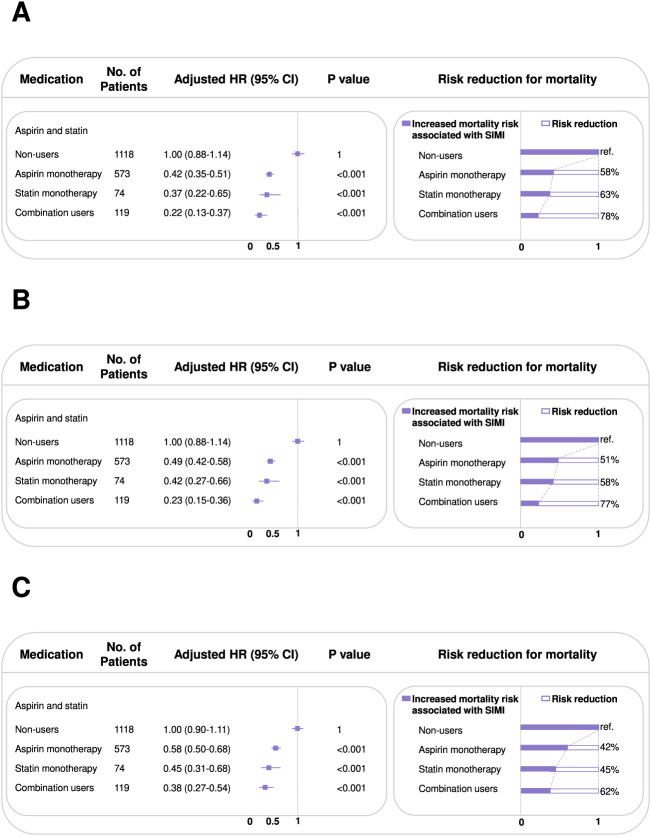
The impact of aspirin monotherapy, statin monotherapy, and combined aspirin and statin therapy on mortality in SIMI patients **(A)** 28-day mortality **(B)** 90-day mortality **(C)** 1-year mortality. HR and 95% CI derived from multivariable Cox proportional hazards models, comparing aspirin monotherapy, statin monotherapy, and aspirin and statin combination against non-users. All models were adjusted for age, gender, body mass index, hypertension, diabetes mellitus, pneumonia, stroke, coronary artery disease, cancer, use of mechanical ventilation or continuous renal replacement therapy, SOFA score, SAPS II score, and lactate level.

Compared to the non-users, the combined users of aspirin and statin conferred a lower risk of 28-day mortality (11.8% vs. 41.9%, HR: 0.22, 95% CI: 0.13–0.37, *P* < 0.001) in the patients with SIMI. The FAR analysis showed that the risk reduction of 28-day mortality was 78% in the combined users, 63% in the statin monotherapy group, and 58% in the aspirin monotherapy group, respectively. A consistent mortality risk reduction pattern was observed across all secondary outcomes of 90-day and 1-year mortality (Figure 2). The PH assumption was tested for each Cox model using Schoenfeld residuals. The global tests yielded non-significant P values for aspirin monotherapy (*P* = 0.095), statin monotherapy (*P* = 0.083), and combination users (*P* = 0.164) when compared with non-users, indicating no violation of the PH assumption. E-value analysis further supported the robustness of the associations, for the combination therapy vs. non-users at 28 days, the E-value was 8.56 (lower bound 4.85). Detailed E-values for all comparisons are provided in Supplementary Table S6. Collinearity diagnostics indicated that all variance inflation factors were within an acceptable range, suggesting no substantial multicollinearity among the covariates.

**FIGURE 3:**
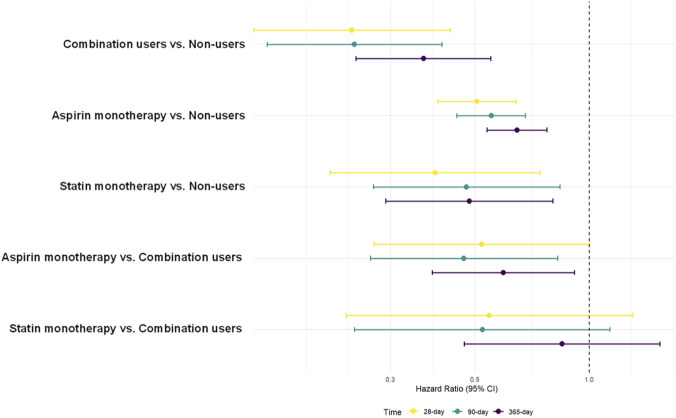
Association of aspirin monotherapy, statin monotherapy, and combination therapy with mortality in the propensity score-matched cohort of SIMI patients. Each row represents a pairwise comparison of different treatment strategies (combination therapy vs. non-users, aspirin monotherapy vs. non-users, statin monotherapy vs. non-users, aspirin monotherapy vs. combination therapy, and statin monotherapy vs. combination therapy). The x-axis displays the HR (95% CI) for mortality at 28-day (yellow), 90-day (teal), and 1-year (purple) follow-up. All analyses were performed in the propensity score-matched cohort to balance baseline confounding variables.

After IPTW adjustment, several covariates remained imbalanced (Supplementary Table S7). Nevertheless, all three treatments were associated with significantly lower mortality at all time points (all *P* < 0.001, Supplementary Table S8). For 28-day mortality, the HR were 0.52 (95% CI: 0.47-0.59) for aspirin monotherapy, 0.44 (95% CI: 0.38-0.51) for statin monotherapy, and 0.40 (95% CI: 0.32-0.49) for combination users. Among the three treatments, combination users consistently showed the lowest HR. The protective effects attenuated over time but remained significant at 1 year, with HR of 0.64 (95% CI: 0.59-0.71) for aspirin, 0.52 (95% CI: 0.46-0.58) for statin, and 0.50 (95% CI: 0.44-0.57) for combination users. In the PSM cohort, we further analyzed the association between combined therapy and prognosis using Cox proportional hazards models (Table 2; Figure 3). For 28-day mortality, the HR were 0.24 (95% CI: 0.13-0.43) for combination users, 0.51 (95% CI: 0.40-0.64) for aspirin monotherapy, and 0.39 (95% CI: 0.21-0.74) for statin monotherapy (all *P* < 0.01). Similar reductions in mortality risk were observed at 90 days and 1 year, although the effect estimates attenuated over time.

**TABLE 2:** Association of aspirin monotherapy, statin monotherapy, and combination therapy with mortality in the propensity score-matched cohort of SIMI patients.

Comparison	Time	HR (95% CI)	P-value
Combination users vs. Non-users	28 days	0.237 (0.131-0.431)	<0.001
	90 days	0.241 (0.142-0.409)	<0.001
	1 year	0.366 (0.243-0.550)	<0.001
Aspirin monotherapy vs. Non-users	28 days	0.506 (0.399-0.641)	<0.001
	90 days	0.552 (0.448-0.679)	<0.001
	1 year	0.645 (0.538-0.774)	<0.001
Statin monotherapy vs. Non-users	28 days	0.393 (0.208-0.741)	0.004
	90 days	0.475 (0.270-0.836)	0.010
	1 year	0.483 (0.291-0.802)	0.005
Combination users vs. Aspirin monotherapy	28 days	0.521 (0.271-1.003)	0.051
	90 days	0.467 (0.265-0.825)	0.009
	1 year	0.594 (0.386-0.914)	0.018
Combination users vs. Statin monotherapy	28 days	0.545 (0.229-1.299)	0.171
	90 days	0.523 (0.241-1.132)	0.100
	1 year	0.848 (0.469-1.532)	0.584

In direct comparisons between the active treatment groups, combined therapy showed a trend toward lower mortality compared with aspirin monotherapy at 28 days (HR 0.52, 95% CI: 0.27-1.00, *P* = 0.05), with significant benefit at 90 days (HR: 0.47, 95% CI: 0.27-0.83, *P* < 0.01) and 1 year (HR: 0.59, 95% CI: 0.39-0.91, *P* = 0.02). No significant differences were observed between combined therapy and statin monotherapy across all time points (HR: 0.55, 95% CI: 0.23-1.30, *P* = 0.17) (Table 2; Figure 3).

**FIGURE 4:**
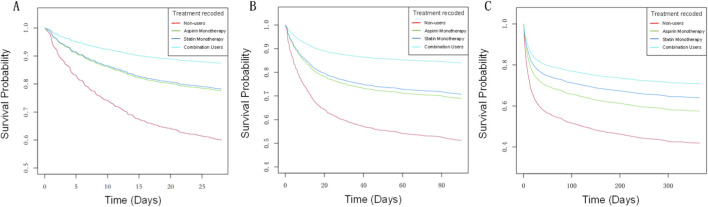
Kaplan–Meier survival curves for 28-day, 90-day, and 1-year mortality in patients with SIMI **(A)** 28-day survival **(B)** 90-day survival **(C)** 1-year survival. The curves represent four treatment groups: non-users (red line), aspirin monotherapy (green line), statin monotherapy (blue line), and aspirin-statin combination therapy (blue-green line).

Kaplan-Meier curves visually confirmed these trends, with the combination users exhibiting consistently superior survival across all timepoints (*P* < 0.001), followed by statin monotherapy group, aspirin monotherapy group (Figure 4). Consistent findings were observed in the IPTW-adjusted Kaplan-Meier curves (Supplementary Figure S2).

### Subgroup analysis

**FIGURE 5:**
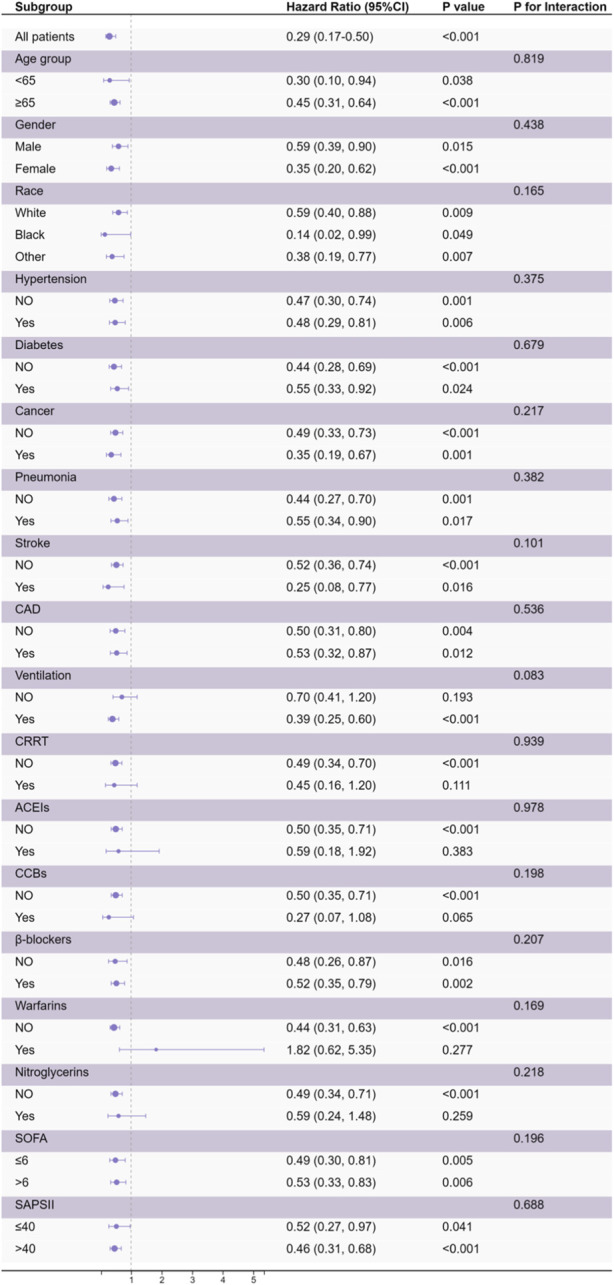
Subgroup analysis of the relationship between combined aspirin and statin therapy and 1-year mortality in SIMI patients. Multivariable Cox proportional hazards models, adjusted for age, gender, body mass index, hypertension, diabetes mellitus, pneumonia, stroke, coronary artery disease, cancer, use of mechanical ventilation or continuous renal replacement therapy, SOFA score, SAPS II score, and lactate level, were fitted within each subgroup. SOFA: Sequential Organ Failure Assessment; SAPS II: Simplified Acute Physiology Score II; CRRT: Continuous Renal Replacement Therapy; ACEI: Angiotensin-Converting Enzyme Inhibitor; ARB: Angiotensin II Receptor Blocker; CCB: Calcium Channel Blocker.

Subgroup analyses were performed according to age, gender, ethnicity, baseline critical illness scores, comorbidities, baseline treatments, and baseline medications. As a result, the baseline clinical characteristics had no interaction on the association between 1-year mortality and the co-medication of aspirin and statin (all *P* for interaction >0.05) (Figure 5).

### External validation

**FIGURE 6:**
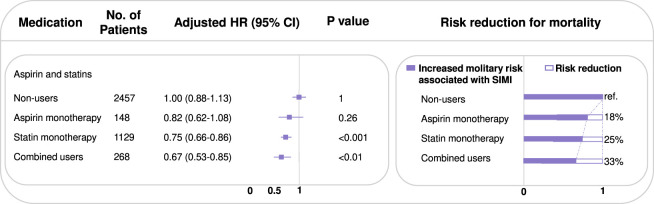
Combined aspirin and statin therapy and in-hospital mortality risk in the external cohort of patients with SIMI. Non-users indicating the SIMI patients without the administration of aspirin or statin was applied as the reference for each comparation. The association between the treatment and in-hospital mortality was evaluated by multivariate Cox regression models with the adjustment of age and gender.

This study, conducted at the Chinese People’s Liberation Army (PLA) General Hospital from January 2020 to October 2025, enrolled 4,002 ICU patients with SIMI to investigate the association between aspirin-statin combination therapy and in-hospital mortality. In the external validation cohort, post-discharge follow-up was not available, so in-hospital mortality was used as the endpoint. Notably, 70% of in-hospital deaths occurred within the first 28 days, and 67.1% of patients had a hospital stay of 28 days or less. Therefore, in-hospital mortality reasonably approximates the 28-day mortality endpoint from the primary analysis. SIMI patients admitted to the ICU were categorized into non-users (n = 2,457), aspirin monotherapy (n = 148), statin monotherapy (n = 1,129), and combination users (n = 268). Baseline characteristics of the patients are presented in Supplementary Table S1. Compared to the non-users, the combination users of aspirin and statin conferred a lower risk of in-hospital mortality (21.6% vs. 24.6%, HR: 0.67, 95% CI: 0.53–0.85, *P* < 0.01) in the patients with SIMI. We employed FAR analysis to examine the relationship between aspirin-statin combination therapy and mortality. FAR analysis demonstrated a 33% reduction in the risk of in-hospital mortality among combination therapy users, compared to a 25% reduction in the statin monotherapy group and a 18% reduction in the aspirin monotherapy group (Figure 6).

### Sensitivity analysis

To assess whether the observed association was robust to the definition of SIMI, we repeated the primary analysis using progressively higher cTnT thresholds (0.03, 0.05, 0.1, and 0.2 ng/mL). As shown in Supplementary Table S10, the hazard ratios for 28-day mortality comparing combination therapy versus non-users remained virtually unchanged across all thresholds (HR ranging from 0.21 to 0.23, all *P* < 0.01). These results indicate that the association between combined aspirin and statin use and lower mortality is not sensitive to the specific cTnT cutoff used to define SIMI.

To address the potential immortal time bias inherent in the exposure definition, we performed a time-dependent Cox regression treating aspirin and statin use as time-varying covariates. As shown in Supplementary Figure S3, the combination of aspirin and statin was significantly associated with lower 28-day mortality after adjusting for potential confounders (HR: 0.544, 95% CI: 0.313-0.945, *P* = 0.031). The individual effects of aspirin (HR: 0.723, 95% CI: 0.508-1.029, *P* = 0.071) and statin (HR: 0.752, 95% CI: 0.486-1.164, *P* = 0.199) did not reach statistical significance. These results suggest that the protective association of combination therapy remains demonstrable even after accounting for timing of medication exposure.

## Discussion

In this study, we observed that combined aspirin and statin therapy was associated with significantly lower mortality in patients with SIMI. Therefore, combined aspirin and statin therapy may represent a promising approach for further investigation in critically ill patients with SIMI.

In this cohort, patients receiving the combination of aspirin and statin had a significantly lower mortality rate at both short-term and long-term follow-up. Previous studies have indicated that monotherapy with either aspirin or statin could reduce mortality in patients with SIMI (Dong et al., 2024; Liu et al., 2025), however, the effect of combination therapy is uncertain. Aspirin and statin may produce combined therapeutic benefits through complementary and synergistic mechanisms. Aspirin acts primarily by inhibiting cyclooxygenase (Mitchell et al., 2021) to limit thrombus formation and uniquely enhances the resolution of inflammation via mediators such as lipoxins (Romano et al., 2015). Statin, beyond cholesterol reduction, improve endothelial function (Van Der Heijden et al., 2008; W et al., 2004) and demonstrate antiplatelet properties (Agarwal et al., 2010; Orsi et al., 2019). Together, their combination constitutes a multi-target intervention that may offer benefits beyond aspirin monotherapy, but no advantage over statin monotherapy was demonstrated in this study, which may be due to the limited sample size of the statin monotherapy group.

We observed notable differences in medication distribution between the two cohorts. In the MIMIC-IV cohort, aspirin monotherapy was more common (30.4%) than statin monotherapy (3.9%), whereas the external cohort showed a lower aspirin use rate (3.7%) but a higher statin use rate (28.2%). This difference may be explained by several clinical practice factors. In Chinese ICUs, clinicians often discontinue pre-existing aspirin upon admission due to concerns about bleeding risks. Although some studies have suggested a potential therapeutic role of aspirin against microthrombosis in sepsis (Carestia et al., 2020; Wang et al., 2018; Trauer et al., 2017), this remains controversial (Almeida et al., 2025). More broadly, aspirin use in the Chinese population is generally lower than that in Western populations (Lu et al., 2019; Wang et al., 2021). These differences in prescribing culture and patient management likely contributed to the observed disparities in medication distribution. Future studies involving multiple healthcare systems and populations are needed to further assess the generalizability of our findings. In contrast to the derivation cohort, aspirin monotherapy did not significantly reduce mortality in the external validation cohort. This discrepancy may be attributed to the relatively small sample size of the aspirin monotherapy group in the validation cohort (n = 148), which limited the statistical power to detect a potentially modest treatment effect. Nevertheless, we still observe a trend towards benefit with aspirin. Despite this variability for monotherapy, the consistent and robust mortality benefit associated with the combination therapy of aspirin and statin across both independent cohorts of SIMI underscores the reliability of the finding.

Although no significant interactions were observed for any of the subgroups, a suggestive trend was noted between ventilated and non-ventilated patients with the combination therapy appeared to be more beneficial in ventilated patients. Although the absence of mechanical ventilation typically indicates less severe illness, consistent treatment effects were observed across all strata of the SOFA and SAPS II in this study. Given previous evidence demonstrating a protective effect of statin in patients with ventilator-associated pneumonia (VAP) (Bruyere et al., 2014), our findings suggest that combination therapy may specifically ameliorate the progression of SIMI triggered by VAP. The underlying mechanisms require further investigation.

This study has several limitations inherent to its retrospective design and data source. Firstly, the primary findings were validated in an external cohort from the Chinese PLA General Hospital, but this validation was limited by the use of in-hospital mortality and adjustment only for age and sex due to data constraints. Moreover, both cohorts are from large tertiary care centers, so generalizability to community hospitals or other populations warrants further investigation. Secondly, the clinical status of critically ill patients is dynamic and could fluctuate with the progression of their illness. The present study, based on retrospective data, provides a cross section of medication use but may not fully capture these temporal changes. Thirdly, the MIMIC-IV database lacks detailed information on drug dosage, treatment duration, reasons for discontinuation, bleeding events, or aspirin contraindications. Therefore, our study could not distinguish between pre-ICU long-term use, new ICU-initiated treatment, or short-term maintenance therapy. This absence precludes assessment of dose-response, adherence, or safety risks like gastrointestinal bleeding, limiting clinical generalizability. Moreover, defining exposure as any aspirin or statin use during the ICU stay mixes different clinical scenarios, including chronic use, new initiation, and late exposure. Without information on timing, dose, duration, or reasons for discontinuation, our findings cannot be interpreted as evidence of treatment efficacy and should be viewed solely as associations generating hypotheses. Fourthly, the definition of exposure may introduce immortal time bias, which limits the interpretation of the observed associations. Although sensitivity analyses including PH testing and E-value calculation indicated that such bias is unlikely to fully account for the observed associations, residual bias cannot be completely ruled out. Therefore, the findings warrant cautious interpretation. Fifthly, the absence of echocardiographic data represents a notable constraint, as it precluded the assessment of cardiac functional status and more precise stratification of myocardial injury severity. Sixth, while our SIMI definition based on a single cTnT peak may be broad, dynamic changes were not analyzable in MIMIC-IV; nonetheless, sensitivity analyses using higher thresholds confirmed the robustness. Finally, although the overall sample size was substantial, certain subgroups such as the combination therapy group remained relatively small, which might affect the statistical power for some comparative analyses. Nevertheless, we employed multivariable regression and FAR analyses to mitigate potential biases and enhance the validity of our conclusions.

## Conclusion

In conclusion, combination therapy with aspirin and statin is associated with reduced mortality in critically ill patients with SIMI.

## Data Availability

The MIMIC-IV database is available from https://mimic.physionet.org. The raw data were extracted using structure query language (SQL) and PostgreSQL, as well as using Excel 2019 and IBM SPSS Statistics for Windows (version 26.0) for data entry and analysis, respectively. The original contributions presented in the study are included in the article/Supplementary Material, further inquiries can be directed to the corresponding author.

## Glossary

SOFA: Sequential Organ Failure Assessment
SAPS II: Simplified Acute Physiology Score II
SIMI: Sepsis-Induced Myocardial Injury
MIMIC: Medical Information Mart for Intensive Care
ICU: Intensive Care Unit
LOS: Length of Stay
SQL: Structured Query Language
HR: Hazard Ratio
CI: Confidence Interval
KM: Kaplan-Meier
FAR: Floating Absolute Risk
ACS: Acute Coronary Syndrome
COPD: Chronic Obstructive Pulmonary Disease
CHF: Chronic Heart Failure
CAD: Coronary Artery Disease
DM: Diabetes Mellitus
MV: Mechanical Ventilation
CRRT: Continuous Renal Replacement Therapy
ACEIs: Angiotensin-Converting Enzyme Inhibitors
ARBs: Angiotensin II Receptor Blockers
β-blockers: Beta-blockers
LMWH: Low Molecular Weight Heparin
cTnT: cardiac Troponin T
WBC: White Blood Cell count
RBC: Red Blood Cell count
PLT: Platelet count
PT: Prothrombin Time
APTT: activated Partial Thromboplastin Time
INR: International Normalized Ratio
CRP: C-Reactive Protein
ALT: Alanine Aminotransferase
AST: Aspartate Aminotransferase
BUN: Blood Urea Nitrogen
SCR: Serum Creatinine
Lac: Lactate
IE: Indirect Effect
DE: Direct Effect
VAP: Ventilator-Associated Pneumonia
CITI: Collaborative Institutional Training Initiative
BIDMC: Beth Israel Deaconess Medical Center
