# Supplementary material for: Effect of combined aspirin and statin therapy on mortality reduction in sepsis-induced myocardial injury
Source: Front Pharmacol. 2026 Jun 9;17:1839835. doi: 10.3389/fphar.2026.1839835 (PMC13286924; doi:10.3389/fphar.2026.1839835)
Supplement: Supplementary file 1 [file Supplementaryfile1.docx]

**Table S1. Baseline characteristics of SIMI patients receiving combined therapy vs. non‑users after PSM (n = 119).**

| **Characteristics*** | **Non-users**  **(n = 119)** | **Combination users**  **(n = 119)** | | | **P-value** | **SMD** |
| --- | --- | --- | --- | --- | --- | --- |
| **Age, mean (SD)** | 78.00 (68.00-86.50) | | 75.00 (67.00-84.00) | | 0.241 | 0.129 |
| **Gender (%)** |  |  | | | 0.237 | 0.067 |
| Female | 74 (62.2%) | 64 (53.8%) | | |  |  |
| Male | 45 (37.8%) | 55 (46.2%) | | |  |  |
| **Ethnicity (%)** |  |  | | | 0.315 | 0.107 |
| White | 86 (72.3%) | 77 (64.7%) | | |  |  |
| Black | 5 (4.2%) | 13 (10.9%) | | |  |  |
| Other | 28 (23.5%) | 29 (24.4%) | | |  |  |
| **Vital signs on admission** |  |  | | |  |  |
| HR (bpm) | 88.79 (18.99) | 83.72 (20.93) | | | 0.052 | 0.174 |
| SBP (mmHg) | 118.50 (105.25-142.00) | 125.00 (103.00-146.00) | | | 0.492 | 0.130 |
| DBP (mmHg) | 64.00 (56.00-74.00) | 62.00 (51.50-76.50) | | | 0.582 | 0.127 |
| Body temperature (◦C) | 36.78 (36.39-37.22) | 36.72 (36.44-37.09) | | | 0.780 | 0.036 |
| RR (bpm) | 20.00 (16.00-23.00) | 18.00 (15.00-22.00) | | | 0.008 | 0.179 |
| SpO_2_ (%) | 98.00 (95.50-100.00) | 98.00 (95.50-100.00) | | | 0.516 | 0.188 |
| **Comorbidity (%)** |  |  | |  | |  |
| Hypertension | 54 (45.4%) | 53 (44.5%) | | | 1.000 | 0.051 |
| DM | 39 (32.8%) | 50 (42.0%) | | | 0.180 | 0.051 |
| Pneumonia | 64 (53.8%) | 44 (37.0%) | | | 0.013 | 0.122 |
| Stroke | 21 (17.6%) | 16 (13.4%) | | 0.474 | | 0.024 |
| CAD | 63 (52.9%) | 62 (52.1%) | | 1.000 | | 0.034 |
| Cancer | 27 (22.7%) | 28 (23.5%) | | 1.000 | | 0.000 |
| **Laboratory examination** |  |  | |  | |  |
| WBC count (10^9^/L) | 11.25 (8.81-16.05) | 11.44 (8.32-14.64) | | 0.620 | | 0.010 |
| RBC count (10^9^/L) | 3.53 (3.09-4.10) | 3.38 (3.05-3.96) | | 0.604 | | 0.012 |
| PLT (10^9^/L) | 180.67 (125.00-233.75) | 182.25 (132.00-238.00) | | 0.800 | | 0.032 |
| PT (s) | 14.05 (12.67-16.28) | 13.85 (12.24-15.01) | | 0.365 | | 0.117 |
| PTT (s) | 31.70 (26.95-36.60) | 31.48 (27.85-38.78) | | 0.667 | | 0.066 |
| INR | 1.25 (1.10-1.47) | 1.20 (1.10-1.35) | | 0.633 | | 0.100 |
| CRP (mg/L) | 74.04 (52.97) | 89.33 (47.70) | | 0.505 | | 0.097 |
| ALT (U/L) | 38.00 (19.12-81.50) | 33.00 (19.00-65.67) | | 0.666 | | 0.097 |
| AST (U/L) | 47.50 (31.00-93.75) | 43.00 (29.00-110.62) | | 0.574 | | 0.111 |
| BUN (mg/dL) | 24.50 (18.25-41.16) | 26.00 (17.50-42.20) | | 0.982 | | 0.123 |
| SCR (mg/dL) | 1.27 (0.90-1.92) | 1.25 (0.88-1.86) | | 0.958 | | 0.046 |
| Lac (mmol/L) | 1.80 (1.20-2.84) | 1.80 (1.30-2.66) | | 0.972 | | 0.002 |
| **Critical assessment on admission** |  |  | |  | |  |
| SOFA score | 6.34 (3.36) | 6.12 (3.76) | | 0.624 | | 0.081 |
| SAPS II score | 53.93 (18.89) | 52.94 (21.89) | | 0.709 | | 0.107 |
| **Treatment (%)** |  |  | |  | |  |
| Ventilation | 80 (67.2%) | 76 (63.9%) | | 0.682 | | 0.069 |
| CRRT | 4 (3.4%) | 10 (8.4%) | | 0.168 | | 0.031 |
| **Medication, n (%)** |  |  | |  | |  |
| ACEI | 13 (10.9%) | 21 (17.6%) | | 0.196 | | 0.139 |
| ARB | 9 (7.6%) | 7 (5.9%) | | 0.785 | | 0.077 |
| Beta-blockers | 71 (59.7%) | 85 (71.4%) | | 0.074 | | 0.119 |
| Oral anticoagulant | 10 (8.4%) | 10 (8.4%) | | 1.000 | | 0.098 |

HR: Heart Rate; SBP: Systolic Blood Pressure; DBP: Diastolic Blood Pressure; RR: Respiratory Rate; SpO₂: Oxygen Saturation; DM: Diabetes Mellitus; CAD: Coronary Artery Disease; WBC: White Blood Cell Count; RBC: Red Blood Cell Count; PLT: Platelet Count; PT: Prothrombin Time; PTT: Partial Thromboplastin Time; INR: International Normalized Ratio; CRP: C-Reactive Protein; ALT: Alanine Aminotransferase; AST: Aspartate Aminotransferase; BUN: Blood Urea Nitrogen; SCR: Serum Creatinine; Lac: Lactate; SOFA: Sequential Organ Failure Assessment; SAPS II: Simplified Acute Physiology Score II; CRRT: Continuous Renal Replacement Therapy; ACEI: Angiotensin-Converting Enzyme Inhibitor; ARB: Angiotensin II Receptor Blocker.

**Table S2. Baseline characteristics of SIMI patients receiving aspirin monotherapy vs. non‑users after PSM** **(n = 551).**

| **Characteristics*** | **Non-users**  **(n = 551)** | **Aspirin monotherapy**  **(n = 551)** | | | **P-value** | **SMD** |
| --- | --- | --- | --- | --- | --- | --- |
| **Age, mean (SD)** | 72.00 (61.00-83.50) | | 71.00 (60.00-82.00) | | 0.436 | 0.033 |
| **Gender (%)** |  |  | | | 0.850 | 0.043 |
| Female | 229 (44.8%) | 233 (45.6%) | | |  |  |
| Male | 282 (55.2%) | 278 (54.4%) | | |  |  |
| **Ethnicity (%)** |  |  | | | 0.450 | 0.000 |
| White | 319 (62.4%) | 289 (56.6%) | | |  |  |
| Black | 58 (11.4%) | 61 (11.9%) | | |  |  |
| Other | 174 (31.6%) | 201 (36.5%) | | |  |  |
| **Vital signs on admission** |  |  | | |  |  |
| HR (bpm) | 91.00 (77.00-105.00) | 89.00 (76.00-102.00) | | | 0.054 | 0.174 |
| SBP (mmHg) | 121.00 (104.00-138.75) | 122.50 (104.75-141.00) | | | 0.321 | 0.058 |
| DBP (mmHg) | 66.00 (55.00-79.00) | 67.50 (54.00-81.00) | | | 0.492 | 0.050 |
| Body temperature (◦C) | 36.72 (36.39-37.22) | 36.83 (36.44-37.22) | | | 0.332 | 0.046 |
| RR (bpm) | 20.00 (16.00-23.00) | 20.00 (16.00-24.00) | | | 0.880 | 0.069 |
| SpO_2_ (%) | 98.00 (95.00-100.00) | 98.00 (95.00-100.00) | | | 0.588 | 0.043 |
| **Comorbidity (%)** |  |  | |  | |  |
| Hypertension | 253 (49.5%) | 258 (50.5%) | | 0.812 | | 0.039 |
| DM | 183 (35.8%) | 187 (36.6%) | | | 0.845 | 0.062 |
| Pneumonia | 266 (52.1%) | 256 (50.1%) | | | 0.573 | 0.016 |
| Stroke | 59 (11.5%) | 66 (12.9%) | | 0.567 | | 0.006 |
| CAD | 124 (24.3%) | 142 (27.8%) | | 0.226 | | 0.072 |
| Cancer | 85 (16.6%) | 80 (15.7%) | | 0.734 | | 0.048 |
| **Laboratory examination** |  |  | |  | |  |
| WBC count (10^9^/L) | 11.78 (8.69-16.31) | 12.45 (8.80-17.04) | | 0.356 | | 0.009 |
| RBC count (10^9^/L) | 3.55 (3.05-4.05) | 3.58 (3.13-4.12) | | 0.278 | | 0.161 |
| PLT (10^9^/L) | 187.75 (131.83-257.00) | 190.00 (138.00-254.00) | | 0.589 | | 0.048 |
| PT (s) | 14.20 (12.84-16.48) | 14.14 (12.50-16.40) | | 0.257 | | 0.059 |
| PTT (s) | 31.15 (27.30-38.40) | 32.09 (27.90-43.15) | | 0.110 | | 0.065 |
| INR | 1.30 (1.13-1.50) | 1.27 (1.10-1.50) | | 0.359 | | 0.036 |
| CRP (mg/L) | 71.20 (34.52-149.50) | 83.50 (28.85-160.10) | | 0.881 | | 0.036 |
| ALT (U/L) | 37.00 (19.00-92.50) | 31.00 (18.00-84.66) | | 0.518 | | 0.052 |
| AST (U/L) | 57.50 (30.50-142.00) | 51.67 (28.25-156.67) | | 0.437 | | 0.052 |
| BUN (mg/dL) | 25.09 (17.00-40.94) | 27.00 (17.00-44.20) | | 0.417 | | 0.010 |
| SCR (mg/dL) | 1.20 (0.85-2.10) | 1.28 (0.85-2.10) | | 0.521 | | 0.049 |
| Lac (mmol/L) | 1.85 (1.20-2.90) | 1.85 (1.30-2.83) | | 0.596 | | 0.084 |
| **Critical assessment on admission** |  |  | |  | |  |
| SOFA score | 6.60 (3.45) | 6.58 (3.67) | | 0.930 | | 0.022 |
| SAPS II score | 56.74 (21.85) | 55.97 (22.53) | | 0.581 | | 0.004 |
| **Treatment (%)** |  |  | |  | |  |
| Ventilation | 323 (63.2%) | 318 (62.2%) | | 0.796 | | 0.020 |
| CRRT | 44 (8.6%) | 45 (8.8%) | | 1.000 | | 0.051 |
| **Medication, n (%)** |  |  | |  | |  |
| ACEI | 44 (8.6%) | 71 (13.9%) | | 0.012 | | 0.182 |
| ARB | 19 (3.7%) | 25 (4.9%) | | 0.455 | | 0.010 |
| Beta-blockers | 252 (49.3%) | 344 (67.3%) | | 0.001 | | 0.135 |
| Oral anticoagulant | 23 (4.5%) | 31 (6.1%) | | 0.329 | | 0.062 |

HR: Heart Rate; SBP: Systolic Blood Pressure; DBP: Diastolic Blood Pressure; RR: Respiratory Rate; SpO₂: Oxygen Saturation; DM: Diabetes Mellitus; CAD: Coronary Artery Disease; WBC: White Blood Cell Count; RBC: Red Blood Cell Count; PLT: Platelet Count; PT: Prothrombin Time; PTT: Partial Thromboplastin Time; INR: International Normalized Ratio; CRP: C-Reactive Protein; ALT: Alanine Aminotransferase; AST: Aspartate Aminotransferase; BUN: Blood Urea Nitrogen; SCR: Serum Creatinine; Lac: Lactate; SOFA: Sequential Organ Failure Assessment; SAPS II: Simplified Acute Physiology Score II; CRRT: Continuous Renal Replacement Therapy; ACEI: Angiotensin-Converting Enzyme Inhibitor; ARB: Angiotensin II Receptor Blocker.

**Table S3. Baseline characteristics of SIMI patients receiving statin monotherapy vs. non‑users after PSM (n = 74).**

| **Characteristics*** | **Non-users**  **(n = 74)** | **Stain monotherapy**  **(n = 74)** | | | **P-value** | **SMD** |
| --- | --- | --- | --- | --- | --- | --- |
| **Age, mean (SD)** | 76.00 (66.25-87.75) | | 74.00 (65.25-80.00) | | 0.254 | 0.080 |
| **Gender (%)** |  |  | | | 0.249 | 0.000 |
| Female | 35 (47.3%) | 43 (58.1%) | | |  |  |
| Male | 39 (52.7%) | 31 (41.9%) | | |  |  |
| **Ethnicity (%)** |  |  | | | 1.000 | 0.096 |
| White | 47 (63.5%) | 49 (66.2%) | | |  |  |
| Black | 6 (8.1%) | 6 (8.1%) | | |  |  |
| Other | 21 (28.4%) | 21 (28.4%) | | |  |  |
| **Vital signs on admission** |  |  | | |  |  |
| HR (bpm) | 93.88 (22.52) | 91.47 (21.59) | | | 0.510 | 0.056 |
| SBP (mmHg) | 122.00 (107.25-137.25) | 111.50 (101.00-133.00) | | | 0.044 | 0.120 |
| DBP (mmHg) | 65.00 (55.75-87.50) | 65.50 (54.25-76.75) | | | 0.269 | 0.089 |
| Body temperature (◦C) | 36.67 (36.44-37.00) | 36.75 (36.44-37.28) | | | 0.244 | 0.136 |
| RR (bpm) | 19.50 (17.00-23.00) | 20.00 (16.00-24.00) | | | 0.646 | 0.015 |
| SpO_2_ (%) | 98.00 (95.25-100.00) | 98.00 (96.00-100.00) | | | 0.420 | 0.108 |
| **Comorbidity (%)** |  |  | |  | |  |
| Hypertension | 33 (44.6%) | 37 (50%) | | 0.600 | | 0.027 |
| DM | 30 (40.5%) | 30 (40.5%) | | | 1.000 | 0.082 |
| Pneumonia | 31 (41.9%) | 27 (36.5%) | | | 0.613 | 0.056 |
| Stroke | 7 (9.5%) | 11 (14.9%) | | 0.451 | | 0.079 |
| CAD | 18 (24.3%) | 14 (18.9%) | | 0.549 | | 0.067 |
| Cancer | 19 (25.7%) | 17 (23.0%) | | 0.848 | | 0.032 |
| **Laboratory examination** |  |  | |  | |  |
| WBC count (10^9^/L) | 11.60 (7.20-18.70) | 12.93 (8.67-17.60) | | 0.415 | | 0.130 |
| RBC count (10^9^/L) | 3.47 (0.59) | 3.49 (0.63) | | 0.869 | | 0.069 |
| PLT (10^9^/L) | 188.00 (139.00-242.00) | 213.33 (153.00-262.00) | | 0.193 | | 0.071 |
| PT (s) | 14.30 (13.17-17.10) | 14.35 (13.20-17.68) | | 0.989 | | 0.112 |
| PTT (s) | 33.20 (27.98-45.10) | 29.20 (26.55-35.17) | | 0.051 | | 0.106 |
| INR | 1.27 (1.15-1.56) | 1.30 (1.20-1.60) | | 0.604 | | 0.154 |
| CRP (mg/L) | 34.60 (23.60-145.45) | 49.20 (35.42-86.28) | | 0.857 | | 0.035 |
| ALT (U/L) | 37.25 (19.25-124.67) | 24.00 (15.00-37.00) | | 0.102 | | 0.118 |
| AST (U/L) | 79.25 (24.75-161.88) | 31.00 (22.00-85.00) | | 0.066 | | 0.150 |
| BUN (mg/dL) | 23.33 (17.62-42.50) | 27.84 (17.69-40.75) | | 0.541 | | 0.048 |
| SCR (mg/dL) | 1.20 (0.84-1.94) | 1.35 (0.91-1.98) | | 0.386 | | 0.109 |
| Lac (mmol/L) | 2.00 (1.60-2.84) | 1.80 (1.31-2.85) | | 0.493 | | 0.108 |
| **Critical assessment on admission** |  |  | |  | |  |
| SOFA score | 6.39 (3.20) | 6.07 (3.18) | | 0.537 | | 0.093 |
| SAPS II score | 52.88 (18.36) | 50.95 (15.59) | | 0.491 | | 0.105 |
| **Treatment (%)** |  |  | |  | |  |
| Ventilation | 37 (50.0%) | 42 (56.8%) | | 0.510 | | 0.163 |
| CRRT | 2 (2.7%) | 4 (5.4%) | | 0.677 | | 0.155 |
| **Medication, n (%)** |  |  | |  | |  |
| ACEI | 5 (6.8%) | 11 (14.9%) | | 0.188 | | 0.117 |
| ARB | 1 (1.4%) | 5 (6.8%) | | 0.204 | | 0.000 |
| Beta-blockers | 33 (44.6%) | 41 (55.4%) | | 0.237 | | 0.190 |
| Oral anticoagulant | 4 (5.4%) | 7 (9.5%) | | 0.251 | | 0.186 |

HR: Heart Rate; SBP: Systolic Blood Pressure; DBP: Diastolic Blood Pressure; RR: Respiratory Rate; SpO₂: Oxygen Saturation; DM: Diabetes Mellitus; CAD: Coronary Artery Disease; WBC: White Blood Cell Count; RBC: Red Blood Cell Count; PLT: Platelet Count; PT: Prothrombin Time; PTT: Partial Thromboplastin Time; INR: International Normalized Ratio; CRP: C-Reactive Protein; ALT: Alanine Aminotransferase; AST: Aspartate Aminotransferase; BUN: Blood Urea Nitrogen; SCR: Serum Creatinine; Lac: Lactate; SOFA: Sequential Organ Failure Assessment; SAPS II: Simplified Acute Physiology Score II; CRRT: Continuous Renal Replacement Therapy; ACEI: Angiotensin-Converting Enzyme Inhibitor; ARB: Angiotensin II Receptor Blocker.

**Table S4. Baseline characteristics of SIMI patients receiving aspirin monotherapy vs. combination users after PSM (n = 117).**

| **Characteristics*** | **Aspirin monotherapy**  **(n = 117)** | **Combination users**  **(n = 117)** | | | **P-value** | **SMD** |
| --- | --- | --- | --- | --- | --- | --- |
| **Age, mean (SD)** | 78.00 (67.00-84.00) | | 75.00 (67.00-84.00) | | 0.346 | 0.011 |
| **Gender (%)** |  |  | | | 0.695 | 0.171 |
| Female | 58 (49.6%) | 62 (53.0%) | | |  |  |
| Male | 59 (50.4%) | 55 (47.0%) | | |  |  |
| **Ethnicity (%)** |  |  | | | 0.822 | 0.176 |
| White | 77 (65.8%) | 76 (65%) | | |  |  |
| Black | 9 (7.7%) | 13 (11.1%) | | |  |  |
| Other | 31 (26.5%) | 28 (23.9%) | | |  |  |
| **Vital signs on admission** |  |  | | |  |  |
| HR (bpm) | 88.52 (18.94) | 83.70 (21.10) | | | 0.067 | 0.118 |
| SBP (mmHg) | 126.50 (102.75-142.50) | 125.00 (104.00-146.00) | | | 0.920 | 0.038 |
| DBP (mmHg) | 63.50 (54.00-77.25) | 63.00 (52.00-77.00) | | | 0.578 | 0.027 |
| Body temperature (◦C) | 36.67 (36.39-37.11) | 36.72 (36.44-37.06) | | | 0.500 | 0.110 |
| RR (bpm) | 20.00 (16.00-23.00) | 18.00 (15.00-22.00) | | | 0.013 | 0.176 |
| SpO_2_ (%) | 98.00 (95.00-100.00) | 98.00 (95.00-100.00) | | | 0.420 | 0.093 |
| **Comorbidity (%)** |  |  | |  | |  |
| Hypertension | 58 (49.6%) | 53 (45.3%) | | 0.604 | | 0.084 |
| DM | 41 (35.0%) | 50 (42.7%) | | | 0.283 | 0.085 |
| Pneumonia | 41 (35.0%) | 44 (37.6%) | | | 0.786 | 0.017 |
| Stroke | 17 (14.5%) | 16 (13.7%) | | 1.000 | | 0.000 |
| CAD | 55 (47.0%) | 60 (51.3%) | | 0.601 | | 0.017 |
| Cancer | 23 (19.7%) | 26 (22.2%) | | 0.748 | | 0.020 |
| **Laboratory examination** |  |  | |  | |  |
| WBC count (10^9^/L) | 11.78 (8.38-15.38) | 11.44 (8.46-14.59) | | 0.687 | | 0.040 |
| RBC count (10^9^/L) | 3.52 (3.15-4.12) | 3.38 (3.08-3.96) | | 0.329 | | 0.065 |
| PLT (10^9^/L) | 185.33 (127.00-238.75) | 184.67 (133.25-239.12) | | 0.944 | | 0.041 |
| PT (s) | 14.40 (12.52-16.82) | 13.84 (12.22-15.05) | | 0.100 | | 0.004 |
| PTT (s) | 32.42 (28.44-46.56) | 31.48 (27.62-39.27) | | 0.250 | | 0.144 |
| INR | 1.30 (1.10-1.52) | 1.20 (1.10-1.33) | | 0.153 | | 0.116 |
| CRP (mg/L) | 85.81 ± 71.98 | 89.33 ± 47.70 | | 0.911 | | 0.184 |
| ALT (U/L) | 35.00 (22.00-72.50) | 32.00 (19.00-65.84) | | 0.570 | | 0.132 |
| AST (U/L) | 58.00 (35.00-107.75) | 43.00 (29.00-114.25) | | 0.232 | | 0.198 |
| BUN (mg/dL) | 26.50 (16.81-41.85) | 25.50 (17.00-42.00) | | 0.851 | | 0.035 |
| SCR (mg/dL) | 1.07 (0.80-2.07) | 1.25 (0.87-1.83) | | 0.356 | | 0.123 |
| Lac (mmol/L) | 1.77 (1.40-2.63) | 1.80 (1.30-2.71) | | 0.906 | | 0.062 |
| **Critical assessment on admission** |  |  | |  | |  |
| SOFA score | 6.16 (3.69) | 6.07 (3.18) | | 0.537 | | 0.079 |
| SAPS II score | 53.27 (22.65) | 53.10 (22.04) | | 0.953 | | 0.084 |
| **Treatment (%)** |  |  | |  | |  |
| Ventilation | 64 (54.7%) | 75 (64.1%) | | 0.183 | | 0.052 |
| CRRT | 6 (5.1%) | 10 (8.5%) | | 0.437 | | 0.030 |
| **Medication, n (%)** |  |  | |  | |  |
| ACEI | 23 (19.7%) | 21 (17.9%) | | 0.874 | | 0.045 |
| ARB | 6 (5.1%) | 7 (6%) | | 1.000 | | 0.035 |
| Beta-blockers | 84 (71.8%) | 83 (70.9%) | | 1.000 | | 0.000 |
| Oral anticoagulant | 8 (6.8%) | 10 (8.5%) | | 0.812 | | 0.064 |

HR: Heart Rate; SBP: Systolic Blood Pressure; DBP: Diastolic Blood Pressure; RR: Respiratory Rate; SpO₂: Oxygen Saturation; DM: Diabetes Mellitus; CAD: Coronary Artery Disease; WBC: White Blood Cell Count; RBC: Red Blood Cell Count; PLT: Platelet Count; PT: Prothrombin Time; PTT: Partial Thromboplastin Time; INR: International Normalized Ratio; CRP: C-Reactive Protein; ALT: Alanine Aminotransferase; AST: Aspartate Aminotransferase; BUN: Blood Urea Nitrogen; SCR: Serum Creatinine; Lac: Lactate; SOFA: Sequential Organ Failure Assessment; SAPS II: Simplified Acute Physiology Score II; CRRT: Continuous Renal Replacement Therapy; ACEI: Angiotensin-Converting Enzyme Inhibitor; ARB: Angiotensin II Receptor Blocker.

**Table S5. Baseline characteristics of SIMI patients receiving statin monotherapy vs. combination users after PSM (n = 71).**

| **Characteristics*** | **Statin monotherapy**  **(n = 71)** | **Combination users**  **(n = 71)** | | | **P-value** | **SMD** |
| --- | --- | --- | --- | --- | --- | --- |
| **Age, mean (SD)** | 74.00 (65.00-80.00) | | 74.00 (67.00-84.50) | | 0.402 | 0.072 |
| **Gender (%)** |  |  | | | 0.131 | 0.091 |
| Female | 42 (59.2%) | 32 (45.1%) | | |  |  |
| Male | 29 (40.8%) | 39 (54.9%) | | |  |  |
| **Ethnicity (%)** |  |  | | | 0.506 | 0.177 |
| White | 46 (64.8%) | 44 (62%) | | |  |  |
| Black | 6 (8.5%) | 9 (12.7%) | | |  |  |
| Other | 19 (26.8%) | 18 (25.4%) | | |  |  |
| **Vital signs on admission** |  |  | | |  |  |
| HR (bpm) | 91.79 (21.93) | 84.25 (21.20) | | | 0.039 | 0.162 |
| SBP (mmHg) | 112.00 (101.00-133.50) | 123.00 (102.00-146.00) | | | 0.146 | 0.126 |
| DBP (mmHg) | 65.00 (54.50-77.50) | 67.00 (54.00-79.00) | | | 0.941 | 0.149 |
| Body temperature (◦C) | 36.72 (36.41-37.20) | 36.83 (36.50-37.14) | | | 0.837 | 0.200 |
| RR (bpm) | 20.00 (16.00-24.00) | 18.00 (15.50-22.00) | | | 0.103 | 0.185 |
| SpO_2_ (%) | 98.00 (96.00-100.00) | 98.00 (95.00-100.00) | | | 0.454 | 0.129 |
| **Comorbidity (%)** |  |  | |  | |  |
| Hypertension | 35 (49.3%) | 34 (47.9%) | | 1.000 | | 0.152 |
| DM | 29 (40.8%) | 29 (40.8%) | | | 0.283 | 0.061 |
| Pneumonia | 41 (35.0%) | 44 (37.6%) | | | 1.000 | 0.126 |
| Stroke | 11 (15.5%) | 8 (11.3%) | | 0.622 | | 0.128 |
| CAD | 14 (19.7%) | 14 (19.7%) | | 1.000 | | 0.072 |
| Cancer | 16 (22.5%) | 15 (21.1%) | | 1.000 | | 0.071 |
| **Laboratory examination** |  |  | |  | |  |
| WBC count (10^9^/L) | 12.95 (8.71-17.85) | 11.72 (9.09-15.38) | | 0.458 | | 0.071 |
| RBC count (10^9^/L) | 3.59 (3.01-3.94) | 3.50 (3.09-3.98) | | 0.661 | | 0.113 |
| PLT (10^9^/L) | 216.00 (153.50-268.75) | 182.25 (133.25-231.88) | | 0.043 | | 0.058 |
| PT (s) | 14.40 (13.20-17.54) | 13.82 (12.00-14.70) | | 0.006 | | 0.180 |
| PTT (s) | 30.45 (26.45-35.35) | 31.40 (27.10-37.95) | | 0.363 | | 0.175 |
| INR | 1.30 (1.20-1.60) | 1.20 (1.10-1.30) | | 0.003 | | 0.197 |
| CRP (mg/L) | 83.83 (72.62) | 85.73 (54.50) | | 0.970 | | 0.120 |
| ALT (U/L) | 24.50 (16.25-57.75) | 37.25 (20.88-75.25) | | 0.043 | | 0.129 |
| AST (U/L) | 32.50 (22.62-88.75) | 52.25 (30.12-118.88) | | 0.078 | | 0.060 |
| BUN (mg/dL) | 28.00 (18.29-41.50) | 24.00 (17.00-38.75) | | 0.315 | | 0.042 |
| SCR (mg/dL) | 1.30 (0.90-2.07) | 1.10 (0.84-1.70) | | 0.199 | | 0.065 |
| Lac (mmol/L) | 1.80 (1.36-2.92) | 1.80 (1.30-2.87) | | 0.717 | | 0.082 |
| **Critical assessment on admission** |  |  | |  | |  |
| SOFA score | 6.13 (3.21) | 6.20 (4.09) | | 0.909 | | 0.031 |
| SAPS II score | 51.07 (15.78) | 53.58 (22.47) | | 0.433 | | 0.092 |
| **Treatment (%)** |  |  | |  | |  |
| Ventilation | 39 (54.9%) | 47 (66.2%) | | 0.229 | | 0.031 |
| CRRT | 4 (5.6%) | 5 (7.0%) | | 1.000 | | 0.060 |
| **Medication, n (%)** |  |  | |  | |  |
| ACEI | 10 (14.1%) | 13 (18.3%) | | 0.654 | | 0.040 |
| ARB | 5 (7%) | 5 (7%) | | 1.000 | | 0.060 |
| Beta-blockers | 39 (54.9%) | 45 (63.4%) | | 0.416 | | 0.093 |
| Oral anticoagulant | 7 (9.9%) | 4 (5.6%) | | 0.537 | | 0.156 |

HR: Heart Rate; SBP: Systolic Blood Pressure; DBP: Diastolic Blood Pressure; RR: Respiratory Rate; SpO₂: Oxygen Saturation; DM: Diabetes Mellitus; CAD: Coronary Artery Disease; WBC: White Blood Cell Count; RBC: Red Blood Cell Count; PLT: Platelet Count; PT: Prothrombin Time; PTT: Partial Thromboplastin Time; INR: International Normalized Ratio; CRP: C-Reactive Protein; ALT: Alanine Aminotransferase; AST: Aspartate Aminotransferase; BUN: Blood Urea Nitrogen; SCR: Serum Creatinine; Lac: Lactate; SOFA: Sequential Organ Failure Assessment; SAPS II: Simplified Acute Physiology Score II; CRRT: Continuous Renal Replacement Therapy; ACEI: Angiotensin-Converting Enzyme Inhibitor; ARB: Angiotensin II Receptor Blocker.

**Table S6.** **E‑values for mortality reduction with aspirin monotherapy, statin monotherapy, and combination therapy versus non‑users in patients with SIMI.**

| **Follow‑up** | **Treatment group vs. non‑users** | **E‑value**  **(point estimate)** | **E‑value range** |
| --- | --- | --- | --- |
| **28 days** | Aspirin monotherapy | 4.19 | (3.33 – 5.16) |
|  | Statin monotherapy | 4.85 | (2.45 – 8.56) |
|  | Combined users | 8.56 | (4.85 – 14.87) |
| **90 days** | Aspirin monotherapy | 3.50 | (2.84 – 4.19) |
|  | Statin monotherapy | 4.19 | (2.40 – 6.87) |
|  | Combined users | 8.16 | (5.00 – 12.81) |
| **1 year** | Aspirin monotherapy | 2.84 | (2.30 – 3.41) |
|  | Statin monotherapy | 3.87 | (2.30 – 5.91) |
|  | Combined users | 4.70 | (3.11 – 6.87) |

E‑values quantify the minimum strength of association that an unmeasured confounder would need to have with both the treatment and the outcome to fully explain the observed hazard ratio, assuming no other biases.

**Table S7.** **Post‑IPTW covariate balance assessment with standardized mean differences identifying imbalanced variables.**

| **Variable** | **SMD** | **Balanced** |
| --- | --- | --- |
| Age | 0.263 | No |
| Gender | 0.097 | Yes |
| DM | 0.175 | No |
| Pneumonia | 0.142 | No |
| Stroke | 0.034 | Yes |
| CAD | 0.062 | Yes |
| Cancer | 0.045 | Yes |
| SOFA Score | 0.311 | No |
| SAPS II Score | 0.388 | No |
| Ventilation | 0.133 | No |
| CRRT | 0.071 | Yes |
| Lac | 0.578 | No |
| SCR | 0.259 | No |
| WBC | 0.202 | No |

DM: Diabetes Mellitus; CAD: Coronary Artery Disease; WBC: White Blood Cell Count; SCR: Serum Creatinine; Lac: Lactate; SOFA: Sequential Organ Failure Assessment; SAPS II: Simplified Acute Physiology Score II; CRRT: Continuous Renal Replacement Therapy.

**Table S8.** **The impact of aspirin monotherapy, statin monotherapy, and combination therapy on mortality in SIMI patients** **after IPTW adjustment.**

| **Medication** | **28-day mortality** | | **90-day mortality** | | **1-year mortality** | |
| --- | --- | --- | --- | --- | --- | --- |
|  | **HR**  **(95% CI)** | **P value** | **HR**  **(95% CI)** | **P value** | **HR**  **(95% CI)** | **P value** |
| **Non-users** | Ref |  | Ref |  | Ref |  |
| **Aspirin monotherapy** | 0.52 (0.47-0.59) | ＜0.001 | 0.57 (0.51-0.63) | ＜0.001 | 0.64 (0.59-0.71) | ＜0.001 |
| **Statin monotherapy** | 0.44 (0.38-0.51) | ＜0.001 | 0.51 (0.45-0.58) | ＜0.001 | 0.52 (0.46-0.58) | ＜0.001 |
| **Combination users** | 0.40 (0.32-0.49) | ＜0.001 | 0.38 (0.31-0.45) | ＜0.001 | 0.50 (0.44-0.57) | ＜0.001 |

IPTW‑adjusted HR (95% CI) and P value for mortality comparing each treatment (aspirin monotherapy, statin monotherapy, combination users) with non‑users. Covariates included age, gender, BMI, diabetes mellitus, pneumonia, stroke, coronary artery disease, cancer, liver cirrhosis, SOFA score, SAPS II score, mechanical ventilation, CRRT, and lactate level. Results are presented for 28‑day, 90‑day, and 1-year follow‑up. All comparisons showed *P* < 0.001.

**Table S9. Baseline characteristics of patients with SIMI in the validation cohort. (n=4,002)**

| **Characteristics*** | | **All Patients (n = 4,002)** | | **Non-users (n = 2,457)** | | **Aspirin** **Monotherapy (n = 148)** | | **Statin Monotherapy (n = 1,129)** | | **Combined Users (n = 268)** |
| --- | --- | --- | --- | --- | --- | --- | --- | --- | --- | --- |
| **Age, mean (SD)** | 66.38 ± 18.78 | | 65.01 ± 18.92 | | 69.64 ± 16.10 | | 67.13 ± 19.61 | | 71.51 ± 13.79 | |
| **Gender (%)** |  | |  | |  | |  | |  | |
| Female | 1,313 (32.81%) | | 797 (32.44%) | | 34 (22.97%) | | 398 (35.25%) | | 84 (31.34%) | |
| Male | 2,689 (67.19%) | | 1,660 (67.56%) | | 114 (77.03%) | | 731 (64.75%) | | 184 (68.66%) | |
| **Comorbidity (%)** |  | |  | |  | |  | |  | |
| Hypertension | 1,438 (35.93%) | | 790 (32.15%) | | 78 (52.70%) | | 409 (36.23%) | | 161 (60.07%) | |
| DM | 786 (19.64%) | | 431 (17.54%) | | 54 (36.49%) | | 209 (18.51%) | | 92 (34.33%) | |
| CAD | 796 (19.89%) | | 352 (14.33%) | | 70 (47.30%) | | 214 (18.95%) | | 160 (59.70%) | |
| **Laboratory examination** |  | |  | |  | |  | |  | |
| WBC count (10^9^/L) | 9.41 (8.03-9.87) | | 9.41 (8.11-9.87) | | 9.16 (7.42-9.87) | | 9.51 (8.61-9.87) | | 9.22 (7.96-9.84) | |
| RBC count (10^9^/L) | 3.69 (3.21-4.24) | | 3.63 (3.18-4.17) | | 3.96 (3.50-4.68) | | 3.72 (3.26-4.24) | | 3.92 (3.39-4.51) | |
| PLT (10^9^/L) | 104.00 (88.00-261.00) | | 97.00 (85.00-244.25) | | 183.00 (97.00-316.00) | | 98.00 (91.00-262.00) | | 144.00 (94.25-266.75) | |
| PT (s) | 17.70 (15.20-24.10) | | 18.00 (15.40-25.70) | | 15.70 (14.33-19.38) | | 17.60 (15.30-23.17) | | 16.90 (14.90-21.52) | |
| INR | 1.47 (1.21-2.20) | | 1.49 (1.22-2.29) | | 1.27 (1.13-1.68) | | 1.44 (1.22-2.06) | | 1.37 (1.16-1.87) | |
| CRP (mg/L) | 7.71 (3.30-9.45) | | 7.79 (3.54-9.45) | | 6.57 (1.21-8.89) | | 7.80 (3.25-9.45) | | 5.38 (1.08-8.71) | |
| ALT (U/L) | 25.30 (9.30-82.00) | | 28.20 (9.40-84.40) | | 20.40 (9.10-81.97) | | 9.90 (9.20-79.35) | | 17.30 (9.30-76.73) | |
| AST (U/L) | 54.40 (21.90-88.30) | | 58.40 (24.80-89.50) | | 37.40 (11.50-87.25) | | 54.40 (19.20-86.40) | | 46.90 (16.10-83.70) | |
| BUN (mg/dL) | 9.60 (8.28-17.09) | | 9.55 (8.21-16.14) | | 9.61 (7.90-21.11) | | 9.60 (8.80-10.91) | | 9.67 (8.67-22.31) | |
| SCR (μmol/L) | 96.40 (80.90-263.50) | | 95.70 (78.20-251.83) | | 99.60 (88.20-426.75) | | 95.90 (82.10-239.10) | | 99.60 (88.10-377.15) | |
| Lac (mmol/L) | 3.43 (2.19-6.83) | | 3.38 (2.14-7.09) | | 3.31 (2.07-6.60) | | 3.44 (2.25-6.10) | | 3.70 (2.38-6.55) | |
| **Critical assessment on admission** |  | |  | |  | |  | |  | |
| SOFA score | 5.00 (3.00-7.00) | | 5.00 (3.00-7.00) | | 5.00 (4.00-7.00) | | 5.00 (3.00-7.00) | | 5.00 (3.00-7.00) | |
| Charlson Score | 2.00 (1.00-3.00) | | 2.00 (1.00-3.00) | | 2.00 (2.00-3.00) | | 2.00 (1.00-3.00) | | 2.00 (2.00-4.00) | |

WBC: White Blood Cell count, RBC: Red Blood Cell count, PLT: Platelet count, PT: Prothrombin Time, INR: International Normalized Ratio, CRP: C-Reactive Protein, ALT: Alanine Aminotransferase, AST: Aspartate Aminotransferase, BUN: Blood Urea Nitrogen, SCR: Serum Creatinine, Lac: Lactate, SOFA score: Sequential Organ Failure Assessment score, and Charlson Score: Charlson Comorbidity Index.

**Figure S1. Directed acyclic graph (DAG) for the study.** Directed acyclic graph (DAG) depicting the causal pathways and confounding structure in the analysis of combined aspirin and statin therapy and mortality. The diagram includes patient demographic factors (age, gender, BMI), comorbid conditions (hypertension [HTN], diabetes mellitus [DM], coronary artery disease [CAD], stroke, pneumonia), the exposure of interest (combined therapy of aspirin and statin), severity scores (SOFA, SAPS II), laboratory markers (lactate [Lac], platelets [PLT], serum creatinine [SCR], white blood cell count [WBC]), supportive interventions (continuous renal replacement therapy [CRRT], mechanical ventilation), and the outcome of mortality. Arrows represent hypothesized direct causal effects between variables.

**Figure S2. Kaplan–Meier survival curves for 28-day, 90-day, and 1-year mortality in patients with SIMI after IPTW adjustment**. (A) 28-day survival. (B) 90-day survival. (C) 1-year survival. The curves represent four treatment groups: non-users (gray line), aspirin monotherapy (blue line), statin monotherapy (green line), and aspirin-statin combination therapy (red line). Covariates included age, gender, BMI, diabetes mellitus, pneumonia, stroke, coronary artery disease, cancer, liver cirrhosis, SOFA score, SAPS II score, mechanical ventilation, CRRT, and lactate level.

**Table S10. Sensitivity analysis of the association between combination therapy and 28‑day** **mortality using different cTnT thresholds for SIMI definition.**

| **Definition of SIMI** | **N** | **HR (95% CI) for 28‑day mortality** | **P value** |
| --- | --- | --- | --- |
| Primary definition (cTnT >0.01 ng/mL) | 119 | 0.22 (0.12-0.41) | ＜0.001 |
| cTnT >0.03 ng/mL | 89 | 0.22 (0.11-0.43) | ＜0.001 |
| cTnT >0.05 ng/mL | 64 | 0.23 (0.11-0.46) | ＜0.001 |
| cTnT >0.1 ng/mL | 42 | 0.21 (0.09-0.51) | ＜0.001 |
| cTnT >0.2 ng/mL | 25 | 0.21 (0.07-0.67) | 0.009 |

 For each threshold, patients with a peak cTnT value exceeding the specified level within 24 hours of ICU admission were included. The table reports the number of combination therapy users (N) under each definition, along with the hazard ratio (HR) and 95% confidence interval (CI) for 28‑day all‑cause mortality comparing combination therapy versus non‑users. Multivariable Cox regression models were adjusted for age, gender, BMI, hypertension, diabetes mellitus, pneumonia, stroke, coronary artery disease, cancer, mechanical ventilation, CRRT, SOFA score, SAPS II score, and lactate level.

**Figure S3. Time‑dependent Cox regression for 28‑day mortality.** Hazard ratios (HR) and 95% confidence intervals (CI) for aspirin monotherapy, statin monotherapy, and combination therapy with 28‑day all‑cause mortality, treating medication exposure as time‑varying covariates. Models were adjusted for age, gender, BMI, hypertension, diabetes mellitus, pneumonia, stroke, coronary artery disease, cancer, mechanical ventilation, CRRT, SOFA score, SAPS II score, and lactate level.
